# microRNA-944 overexpression is a biomarker for poor prognosis of advanced cervical cancer

**DOI:** 10.1186/s12885-019-5620-6

**Published:** 2019-05-06

**Authors:** Sunyoung Park, Jungho Kim, Kiyoon Eom, Sehee Oh, Sunghyun Kim, Geehyuk Kim, Sungwoo Ahn, Kwang Hwa Park, Dawn Chung, Hyeyoung Lee

**Affiliations:** 1 Department of Biomedical Laboratory Science, College of Health Sciences, Yonsei University, 1 Yonseidae-gil, Wonju-si, Gangwon-do 26493 Republic of Korea; 2 Department of Clinical Laboratory Science, College of Health Sciences, Catholic University of Pusan, Pusan, South Korea; 3 Department of Pathology, Yonsei University, Wonju College of Medicine, Wonju, South Korea; 4 Department of Obstetrics and Gynecology, Gangnam Severance Hospital, Yonsei University College of Medicine, 146-92 Dongok-dong, Gangnam-gu, Seoul, Republic of Korea

**Keywords:** microRNAs, Survival, Prognosis, Uterine cervical neoplasm

## Abstract

**Background:**

One-third of cervical cancer patients are still diagnosed at advanced stages. The five-year survival rate is decreased in about 50% of advanced stage cervical cancer patients worldwide, and the clinical outcomes are remarkably varied and difficult to predict. One of the miRNAs known to be associated with cancer tumorigenesis is miR-944. However, the prognostic value of miR-944 in cervical cancer has not been fully investigated. The aim of this study was to analyze clinical significance and prognostic value of miR-944 in cervical cancer.

**Methods:**

The expression levels of miR-944 were detected using quantitative reverse transcription polymerase chain reaction in five types of cervical cancer cell lines and 116 formalin-fixed paraffin-embedded (FFPE) cervical tissues. The association between the expression levels of miR-944 and prognostic value was analyzed using the Kaplan-Meier analysis and Cox proportional hazards model.

**Results:**

The expression levels of miR-944 in cervical cancer tissues were significantly higher compared with those in normal tissues (*P* < 0.0001). Moreover, the expression levels of miR-944 in cervical cancer cell lines and FFPE tissues with human papillomavirus (HPV) infection were significantly higher compared to those without HPV infection (*P* < 0.01 and *P* = 0.02). High miR-944 expression was also markedly associated with bulky tumor size (*P* = 0.026), advanced International Federation of Gynecology and Obstetrics (FIGO) stage (*P* = 0.042), and lymph node metastasis (*P* = 0.030). In particular, high miR-944 expression group showed shorter overall survival than the low miR-944 expression group in the advanced FIGO stage (84.4% vs. 44.4%, HR = 4.0, and *P* = 0.01).

**Conclusions:**

These results suggest that miR-944 may be used as a novel biomarker for improving prognosis and as a potential therapeutic target.

**Electronic supplementary material:**

The online version of this article (10.1186/s12885-019-5620-6) contains supplementary material, which is available to authorized users.

## Background

Cervical cancer is the fourth most common cancer in women worldwide after cancer of the breast, colon, and lung [1]. According to the World Health Organization (WHO), each year approximately 528,000 new cases are recorded, and 266,000 deaths occur due to cervical cancer [2]. Cervical cancer is mainly caused by infection with high-risk human papillomavirus (HR-HPV) genotypes [3]. With the development of screening and prevention methods of cervical cancer such as HPV co-testing and HPV vaccination, early diagnosis programs of cervical dysplasia and cancer lead to decrease the incidence, morbidity, and mortality of cervical cancer [4].

However, nearly 50% out of all cervical cancer patients worldwide are still diagnosed with stages IB2 to IVA according to International Federation of Gynecology and Obstetrics (FIGO), whereas about one-third of patients diagnosed with cervical cancer in Korea presented locally advanced stage of the disease [5]. Currently, the standard treatment guideline for patients with advanced FIGO stage IIB and more than stage IIB is radiotherapy combined with platinum-based chemotherapy [6]. Unfortunately, these patients have a higher recurrence rate and a worse survival rate in the first 5 years [5]. Therefore, it is of great importance to identify a novel biomarker that can reliably detect cervical cancer and improve clinical monitoring.

microRNAs (miRs or miRNAs), small non-coding RNAs consisting of approximately 22 nucleotides, regulate gene expression by binding to partially or fully complementary sequences in target mRNAs, resulting in translational inhibition or mRNA degradation, respectively [5]. miRNAs have been suggested to function as oncogenes or tumor suppressors based on their function of inhibiting the expression of tumor suppressive or oncogenic target mRNAs, respectively [6–8]. Numerous studies have shown that dysregulation of miRNAs plays an essential role in cell proliferation, cell cycle regulation, differentiation, and apoptosis and is related to various tumors such as colon, gastric, breast, lung, and cervical cancers [9–13].

miR-944 is located in the intron of the tumor protein p63 gene (*TP63*) mapped to chromosome 3q28. Previous reports show that miR-944 functions as an oncogene in a number human cancers, including cervical cancer, endometrial cancer, breast cancer, and lung cancer by promoting cell migration, proliferation, and invasion [14–17]. However, some studies have reported that miR-944 can function as a tumor suppressor, inhibiting migration in colorectal cancer, gastric cancer and breast cancer [18–20]. In cervical cancer, miR-944 promotes cell proliferation and migration in CaSki and HeLa cervical cancer cell lines [16, 21]. To our knowledge, there are no other further studies on the clinical relevance of miR-944 in cervical cancer, and specifically in terms of prognostic value.

Human papillomavirus infection is known to one of the most significant risk factors for cervical cancer [22]. Several studies suggested that the sustained expression of the two oncogenic genes *E6* and *E7* of HPV is involved in cervical cancer progression by degradation of p53 and deactivation of retinoblastoma protein (pRB) [23–27]. Recently, miRNA sequencing data from The Cancer Genome Atlas (TCGA) was reported that miRNA clusters such as miR-205-5p, miR-944, miR-200a-5p, miR-30a-5p, miR-338-3p, miR-224-5p, and miR-193b-3p were associated with cervical cancer and especially miR-944 was shown the significant difference between HPV positive and HPV negative cervical cancer [28]. However, there is no further study of the relationship between miR-944 and HPV E6/E7 expression in cervical cancer.

In this study, the prognostic value of miR-944 was investigated using 116 formalin-fixed paraffin-embedded (FFPE) cervical cancer tissues and normal tissues. Furthermore, the association between miR-944 and HPV E6/E7 mRNA-positive or -negative was explored.

## Methods

### Study population

A total of 66 FFPE cervical cancer tissues and 50 FFPE normal cervical tissues were collected between January 2010 and December 2014. A total of 66 cervical cancer cases data on age, tumor size, FIGO stage, lymph node metastasis, and HPV infection were retrospectively reviewed from patient electrical medical records. The 50 FFPE normal cervical tissues consisted of 44 patients with non-cervical, benign, uterine disease and six cervical cancer patients with tumor-matched non-cancerous tissues (Table 1). This study was approved by the Institutional Ethics Committee of Yonsei University Wonju Severance Christian Hospital (approval no. CR315052), and all subjects provided written informed consent.

**Table 1:** Clinical characteristics

Features	Cervical cancer, N (%)	Normal, N (%)
Total, n (%)	66 (100.0)	50 (100.0)
Age		
< 45 years	20 (30.3)	20 (40.0)
≥ 45 years	46 (69.7)	30 (60.0)
Histological type		
ADC	7 (10.6)	
SCC	59 (89.4)	
Tumor size		
< 4 cm	30 (45.5)	
≥ 4 cm	36 (54.5)	
FIGO stage		
IA-IIA	29 (43.9)	
IIB-IVB	37 (56.1)	
Lymph node metastasis		
No	35 (53.0)	
Yes	31 (47.0)	
HPV E6/E7 expression		
Negative	12 (18.2)	
Positive	54 (81.8)	
Survival		
Alive	52 (78.8)	
Died	14 (21.2)	

Abbreviations: *FIGO* International Federation of Gynecology

### Cell culture

Cervical cancer cell lines C33A (HPV-negative), SiHa (HPV 16), Caski (HPV 16), HeLa (HPV 18), and ME180 (HPV 18, 68) were purchased from the Korean Cell Line Bank (Seoul, Republic of Korea) and the American Type Culture Collection (Manassas, VA, USA). Dulbecco’s modified Eagle’s medium (DMEM) with 10% fetal bovine serum (FBS; Gibco, Carlsbad, CA, USA) and 1% penicillin/streptomycin (Gibco, Carlsbad, CA, USA) was used to maintain the cells in tissue culture and the five cell lines were maintained at 37 °C with 5% CO_2_.

### Deparaffinization of FFPE tissue and total RNA extraction

For total RNA extraction from FFPE cervical tissue, three to four 10-μm-thick sections were transferred to 1.5 mL tube each. Before extracting RNA, the paraffin was removed from the tissue sections by adding 160 μL of deparaffinization solution (Qiagen, Hilden, Germany) followed by incubation for 3 min at 56 °C. Total RNA extraction was extracted according to the manufacturer’s protocol (Qiagen RNeasy FFPE kit, Qiagen). Next, the concentration of the total RNA was measured with an Infinite 200 spectrophotometer (Tecan, Salzburg, Austria). Total RNA was stored at − 80 °C until used.

### microRNA RT-qPCR analysis

We used the TaqMan miRNA Reverse Transcription kit (Applied Biosystems, Foster City, CA, USA) to synthesize cDNA according to the manufacturer’s instructions. Reverse transcription reactions were performed using 10 ng of total RNA and specific reverse transcription primers (Life Technologies) for *Homo sapiens* (hsa)-miR-944 (assay ID 002189), and the internal reference RNU6B (assay ID 001093). Reverse transcription was performed at 16 °C for 30 min, 42 °C for 30 min, 85 °C for 5 min.

After the reverse transcription, quantitative PCR reaction was performed using the TaqMan microRNA assay (Applied Biosystems) according to the manufacturer’s instructions. Briefly, the initiation step of PCR cycling conditions at 95 °C for 10 min, followed by an amplification step of 40 cycles at 95 °C for 15 s and 60 °C for 60 s. The miR-944 expression levels were calculated via the comparative cycle threshold (C_T_) method. The C_T_ values of miR-944 were normalized to the level of RNU6B.

### HPV E6/E7 mRNA RT-qPCR assay

M-MLV reverse transcriptase kit (Invitrogen, Carlsbad, CA, USA) was used for complementary DNA (cDNA) synthesis. Briefly, add 0.25 μg random hexamers, 1 μL 10 mM dNTP mix, and 5 μL DEPC-treated water and 10 μL total RNA. The mixture was then incubated at 65 °C for 5 min and quickly cooled on ice. After that, 4 μL of 5x Buffer, 2 μL of 0.1 M dithiothreitol (DTT) and 1 μL of M-MLV Reverse Transcriptase (RT) were added to the first mix. The reverse transcriptase reaction was carried out at 25 °C for 10 min, at 37 °C for 50 min, and at 70 °C for 15 min.

Detection of HPV E6/E7 mRNA in FFPE cervical tissues was performed by OPTIMYGENE HPV E6/E7 mRNA RT-qDx assay kit (Optipharm M&D, Osong, Republic of Korea), according to the manufacturer’s instructions. The reverse transcription quantitative PCR (RT-qPCR) assay was carried out using the CFX96 system (Bio-Rad, Hercules, CA). Real-time PCR amplification for HPV E6/E7 mRNA was performed in a total volume of 20 μL, containing 10 μL of 2x Thunderbird probe qPCR mix (Toyobo, Osaka, Japan), 5 μL primer and TaqMan probe mixture, 3 μL distilled water and 2 μL template cDNA. Positive and negative controls were contained per each procedure. Reaction conditions of real-time PCR were as follows: 95 °C for 3 min, and 40 cycles of 95 °C for 3 s and 55 °C for 30 s. Glyceraldehyde-3-phosphate dehydrogenase (GAPDH) was used as an endogenous control and confirmation of mRNA degradation.

### The Cancer genome atlas (TCGA) analysis

Raw data for miR-944 and clinical information in cervical cancer were extracted from the TCGA open source repository website (http://firebrowse.org/) on 01/28/2016. Variables, such as follow-up times, tumor stage, race, and reads per million of miR-944 in 180 cervical cancer patients were included. Information of HPV E6/E7-positive and HPV E6/E7-negative cervical cancers matched with patients from TCGA open source repository website was obtained from the Banister et al. study [29]. The HPV E6/E7 expression were classified as HPV alpha-9, HPV alpha-7, and HPV negative cervical cancer based on HPV E6/E7 expression. The reads per million of miR-944 according to HPV E6/E7 expression were analyzed by student t-test.

### Statistical analysis

All statistical analyses were performed using GraphPad Prism version 6 software (La Jolla, CA, USA) and SPSS version 21.0 software (IBM, Armonk, NY, USA). Wilcoxon matched-pairs singed rank test was performed for comparison between the paired cervical normal and cancer tissues. Student’s t-test were conducted for comparison between normal and cancer tissues. To identify whether miR-944 is affected by HPV E6/E7 mRNA positive or negative cervical cancer tissues, the expression levels of miR-944 was compared by student’s t-test. To determine the clinical cut-off value of miR-944 between cervical cancer and normal tissues, receiver operator characteristic (ROC) curve analysis were performed. Based on the clinical cut-off value, cases were divided into two groups according to the expression level of miR-944 (high miR-944 vs. low miR-944). Potential associations between the expression of miR-944 and various prognostic parameters were analyzed by the Chi-square test. Survival was estimated by the Kaplan-Meier method and evaluated by the log-rank test. Multivariate analyses of prognostic values were evaluated using the Cox proportional hazards model. In all analyses, a *P-*value of less than 0.05 was considered statistically significant.

## Results

### miR-944 expression levels in normal and cancerous cervical tissues

**Fig. 1:**
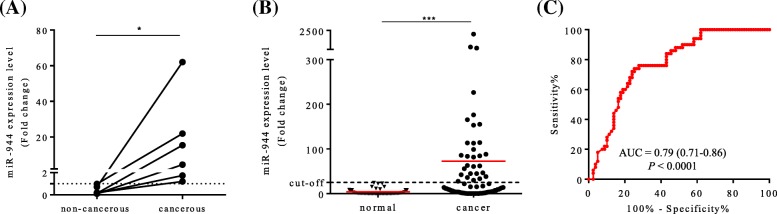
Relative miR-944 expression in FFPE cervical cancer tissues and normal cervical tissues. **a** Relative miR-944 expression in paired samples (*n* = 6). The mean miR-944 expression level in the cervical cancer tissues was significantly higher than the pair-matched non-cancerous cervical tissues (*P* = 0.031). **b** The relative miR-944 expression levels were compared between cervical cancer (*n* = 66) and normal cervical tissues (*n* = 50). The miR-944 expression levels in cervical cancer tissues were significantly higher than those in normal cervical tissues (*P* < 0.0001). **c** Receiver operator characteristic curve analysis for determining cut-off value is distinguishing normal and cancer cells was performed. Optimal cut-off point for miR-944 with an AUC of 0.79 (*P* < 0.0001; 95% confidence interval [CI], 0.71–0.86). * *P* < 0.05, ** *P* < 0.01, and *** *P* < 0.001

To explore the expression levels of miR-944 in cervical cancer cells, six cervical cancer patients with tumor-matched non-cancerous tissues were evaluated by RT-qPCR. The results showed that the expression levels of miR-944 were significantly higher in cervical cancer tissues than those in matched non-cancerous cervical tissues (*n* = 6, *P* = 0.031; Fig. 1a). Subsequently, similar expression levels of miR-944 were observed by RT-qPCR in 66 cervical cancer tissues and 50 normal cervical tissues. The expression levels of miR-944 in the cervical cancer tissues were significantly higher than in normal cervical tissues (*P* < 0.0001; Fig. 1b). The expression level of miR-944 in cervical cancer and normal tissues was plotted as a ROC curve to assess cut-off values for discriminating cervical cancer from normal tissues. At the optimal cut-off point, the area under the curve (AUC) of miR-944 was 0.79 (*P* < 0.0001; 95% confidence interval (CI), 0.71–0.86 (Fig. 1c).

### Association between miR-944 expression and HPV infection status

Since the main cause of cervical cancer is HPV infection, we investigated whether the expression of miR-944 and HPV infection status were related. The expression levels of miR-944 in cervical cancer cell lines with or without HPV infection were compared. Expression levels of miR-944 in the HPV-positive cervical cancer cell lines SiHa (HPV 16-infection), Caski (HPV 16 infection), HeLa (HPV 18 infection) and ME180 (HPV 18, 68 infection) were significantly higher than in the HPV-negative cervical cancer line C33A (*P* < 0.01, *P* < 0.01, *P* < 0.01, and *P* < 0.001, respectively), supporting the hypothesis that HPV infection may affect miR-944 expression levels (Additional file 1).

**Fig. 2:**
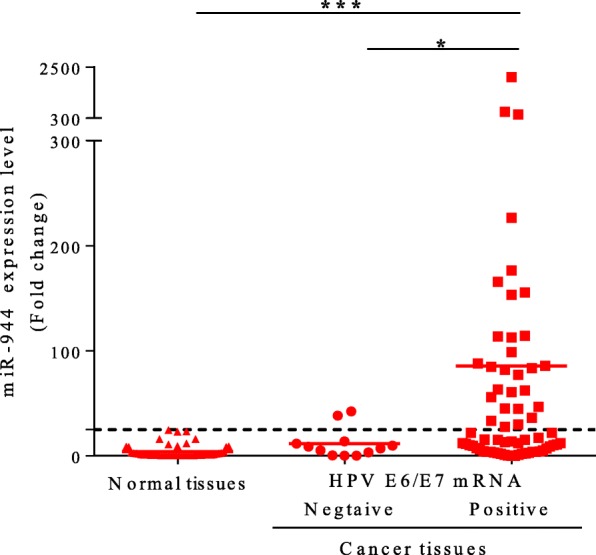
Relative expression of miR-944 in HPV E6/E7 mRNA positive and negative cervical cancer tissues. Comparison of the relative expression level of miR-944 among the HPV E6/E7 mRNA positive cervical cancer tissues (*n* = 67), HPV E6/E7 mRNA negative cervical cancer tissues (*n* = 12), and normal tissues (*n* = 50). The expression level of miR-944 in HPV E6/E7 mRNA positive cervical cancer tissues was significantly higher than in HPV E6/E7 mRNA negative cervical cancer tissues (*P* = 0.02). * *P* < 0.05, ** *P* < 0.01, and *** *P* < 0.001

Next, the expression levels of miR-944 in the 66 FFPE cervical cancer tissues according to HPV infection status were investigated. Among 54 HPV E6/E7 mRNA expression-positive and 12 HPV E6/E7 mRNA expression-negative cancer tissues, the expression levels of miR-944 were significantly up-regulated in HPV E6/E7 mRNA expression-positive cancer tissues (*P* = 0.02; Fig. 2). Furthermore, 171 cervical cancer tissues comprised of samples from various races and HPV types (TCGA data) classified by HPV-E6/E7 based genes were additionally analyzed (Additional file 2). Similarly, the expression levels of miR-944 in TCGA data were also significantly up-regulated in HPV-E6/E7 positive cancer tissues, especially alpha 9 and alpha 7 species (*P* < 0.0001; Additional file 3).

### Associations between miR-944 expression and clinicopathological characteristics

To investigate the association between the miR-944 expression levels and clinicopathological characteristics, Chi-square test was used to analyze data from 66 cervical cancer patients. miR-944 expression levels showed no association with age and histological type (*P* = 0.815 and *P* = 0.174). However, miR-944 expression levels showed significant association with tumor size, FIGO stage, and lymph node metastasis, indicating their usefulness as prognostic parameters (*P* = 0.026, *P* = 0.042, and *P* = 0.030; Table 2). The expression of miR-944 was low in tumors less than 4 cm (76.7%), in the early FIGO stage (75.9%), and in the absence of lymph node metastasis (74.3%). Interestingly, patients with tumors larger than 4 cm, advanced FIGO stage, and lymph node metastasis showed low and high of miR-944 levels at similar percentages (50 and 50% for larger than 4 cm tumors, 51.4 and 48.6% for advanced FIGO stage, and 48.4 and 51.6% for lymph node metastasis). Not only the conventional prognostic parameters but also biological characteristics of miR-944 provide another additional information. Therefore, we further analyzed whether miR-944 is useful as a prognostic marker that can provide additional information to existing prognostic factors.

**Table 2:** Association between the miR-944 expression levels and clinicopathological characteristics of patients with cervical cancer

Features	No. (%)	miR-944 expression		*P*
	(*n* = 66)	Low, n (%)	High, n (%)	
No. of patients	66	41 (62.1)	25 (37.9)	
Age				0.815
< 45 years	20	12 (60.0)	8 (40.0)	
≥ 45 years	46	29 (63.0)	17 (37.0)	
Histological type				0.174
ADC	7	6 (85.7)	1 (12.9)	
SCC	59	35 (59.3)	24 (40.7)	
Tumor size				0.026
< 4 cm	30	23 (76.7)	7 (23.3)	
≥ 4 cm	36	18 (50.0)	18 (50.0)	
FIGO stage				0.042
IA-IIA	29	22 (75.9)	7 (24.1)	
IIB-IVB	37	19 (51.4)	18 (48.6)	
Lymph node metastasis				0.030
No	35	26 (74.3)	9 (25.7)	
Yes	31	15 (48.4)	16 (51.6)	

Abbreviation; *FIGO* International Federation of Gynecology; *P*, Chi-square *p*-value

### Association between miR-944 expression and patient survival

**Fig. 3:**
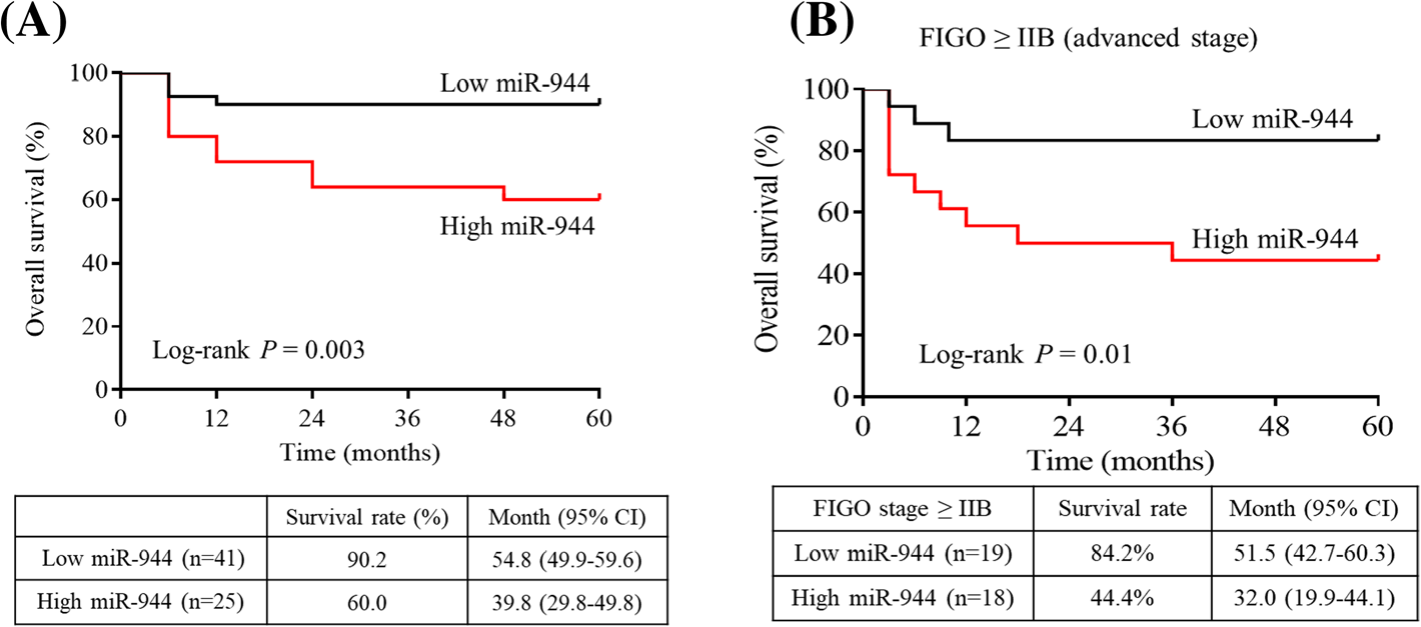
Kaplan-Meier survival curve for patients with cervical cancer according to miR-944 expression. **a** High or low miR-944 expression level were determined using the cut-off value of miR-944 in cervical cancer and normal tissues. High miR-944 expression level group showed poor survival rates compared to low miR-944 expression level group (log-rank test, *P* = 0.003). **b** Among the 37 patients with an advanced FIGO stage, including of 19 patients with low miR-944 expression and 18 with high miR-944 expression, the overall survival rate was 84.2 and 44.4% (log-rank test, *P* = 0.01)

To explore overall survival in patients with high and low expression of miR-944, overall survival time was obtained from the date of the initial surgery to time of death. Kaplan-Meier survival analysis showed poor prognosis with high miR-944 expression (Log-rank test, *P* = 0.003). A 5-year survival rate of 90.2% in patients with low miR-944 expression levels (*n* = 41) ranged from 49.9 to 59.6 months (mean, 54.8 months), whereas 5-year survival rate of 60.0% in patients with high miR-944 expression levels (*n* = 25) ranged from 29.8 to 49.8 months (mean, 39.8 months) (Fig. 3a).

To compare the prognostic parameters and miR-944, which is a newly found prognostic marker, Cox proportional hazards regression was used to investigating the association between survival time of patients and clinical parameters together with miR-944 expression. Using univariate analysis, high miR-944 expression levels and three clinicopathological parameters which were tumor size (HR = 5.6, 95% CI 1.3–25.2, *P* = 0.024), FIGO stage (HR = 11.8, 95% CI 1.5–90.2, *P* = 0.018), and lymph node metastasis (HR = 8.2, 95% CI 1.8–36.5, *P* = 0.006) were associated with poor prognosis of cervical cancer. All significant parameters were further analyzed using multivariate analysis. The results revealed that FIGO stage (HR = 9.1, 95% CI 1.2–71.1, *P* = 0.034) and high miR-944 expression (HR = 3.5, 95% CI 1.1–11.2, *P* = 0.034) could be independent prognostic factors for the patients with cervical cancer (Table 3).

**Table 3:** Univariate and multivariate analysis of prognostic factors together with miR-944 expression in cervical cancer

Features	Overall survival					
	Univariate analysis			Multivariate analysis		
	HR	95% CI	*P*	HR	95% CI	*P*
Ages						
< 45 years vs. ≥ 45 years	2.8	0.6–12.4	0.18			
Histological type						
ADC vs. SCC	1.5	0.2–11.8	0.68			
Tumor size						
< 4 cm vs. ≥ 4 cm	5.6	1.3–25.2	0.024			
FIGO stage						
<IIb vs. ≥ IIb	11.8	1.5–90.2	0.018	9.1	1.2–71.1	0.034
Lymph node metastasis						
no vs. yes	8.2	1.8–36.5	0.006			
miR-944 expression						
Negative vs. Positive	4.7	1.5–15.0	0.009	3.5	1.1–11.2	0.034

Abbreviations: *ADC* Adenocarcinoma, *SCC* Squamous cell carcinoma, *FIGO* International Federation of Gynecology

In an advanced FIGO stage (*n* = 37), Kaplan-Meier survival analysis revealed the poor prognosis of patients with high miR-944 expression levels (Log-rank test, *P* = 0.01). The 5-year survival rate in patients with low miR-944 expression levels (*n* = 19) was 84.2% and ranged from 42.7 to 60.3 months (mean, 51.5 months), whereas the 5-year survival rate in patients with high miR-944 expression levels (*n* = 18) was 44.4% and ranged from 19.9 to 44.1 months (mean, 32.0 months) (Fig. 3b).

## Discussion

In this study, we investigated miR-944 expression levels in FFPE cervical cancer and normal tissues by RT-qPCR methods, and the result showed poor prognosis in patients with high miR-944 expression levels and in an advanced stage of cervical cancer. In addition, we demonstrated that there was an association between high expression levels of miR-944 and expression levels of HPV oncogene E6/E7 in HPV-positive cervical cancer cells by analysis of FFPE cervical cancer tissues and TCGA data.

The expression levels of miR-944 were significantly higher in cervical cancer tissues as compared to those in paired non-cancerous tissues (*n* = 6) (Fig. 1), which suggested that miR-944 plays a role in tumorigenesis through clinical cervical cancer tissues. Xie et al. (2015) also had shown that high miR-944 expression levels are associated with tumorigenesis of cervical cancer, based on analysis of cervical cancer cell lines (Caski and HeLa) [16]. We further examined 66 cervical cancer and 50 normal FFPE samples and showed that the expression of miR-944 in cervical cancer was up-regulated which was in line with previous reports [16].

To gain insight into the clinical significance and prognostic value of miR-944, we investigated the relationship between miR-944 expression levels and the clinicopathological characteristics of cervical cancer patients and found that expression of miR-944 was firmly related to the tumor size, FIGO stage, and lymph node metastasis (Table 2). Ma et al. reported that up-regulation of miR-944 has also been associated with lymph node metastasis and advanced stage of lung squamous cell carcinoma [30].

Furthermore, survival analysis of cervical cancer patients showed that high expression levels of miR-944 were associated with poor survival prognosis (Fig. 3a). As a prognostic marker, FIGO stage and high miR-944 expression levels were shown useful prognostic indicators of poor survival (Table 3). Notably, in advanced cervical cancer, high miR-944 expression levels were significantly poorer prognosis than low miR-944 expression levels (Fig. 3b).

Previous studies have attempted to identify the mRNA target of miR-944 in cervical cancer, and Xie et al. showed that miR-944 has two target genes, HECT domain ligase W2 (HECW2), which are known to regulate p63 stabilization, and S100P binding protein (S100PBP), which is known to reduce adhesion and invasion. miR-944 was predicted to play an oncogenic role in cervical cancer malignancy by repressing these two genes (HECW2 and S100PBP) that function as a tumor suppressors [16].

Several studies have reported that the expression levels of some miRNAs are associated with poor prognosis in cervical cancer, such as downregulation of miR-335 from Wang et al. [31], down-regulation of miR-145 and up-regulation of miR-9 from Azizmohammadi et al. [32]. Recently, Jiang et al. suggested that down-regulation of circulating miR-101 is associated with poor prognosis of cervical cancer [33]. Our study also suggests that high expression of miR-944 could be of clinical relevance for poor prognosis. The miR-944 expression has been analyzed in colorectal, bladder, and breast cancer. High miR-944 expression levels have been associated with tumor recurrence in colorectal cancer [34] and chemo-resistance in bladder cancer [35]. He et al. found that miR-944 is significantly up-regulated in the blood and tumor tissues of breast cancer patients [36]. Like our study, miR-944 is remarkable tumor-associated microRNA.

Interestingly, in our study, miR-944 expression in HPV E6/E7 mRNA-positive cervical cancer was higher than that in HPV E6/E7 mRNA-negative cervical cancer (*P* = 0.02; Fig. 2). Meanwhile, among five cervical cancer cell lines, the expression of miR-944 in cervical cancer cell lines with HPV infection (SiHa and HeLa, Caski, and ME180) was significantly higher than that in the cervical cancer cell line without HPV infection (C33A) (Additional file 1) as well as TCGA data (Additional file 3), analyzed by using individual miR-944 reads and HPV E6/E7 expression status. It suggests that up-regulation of miR-944 is associated with HPV infection and the carcinogenic processes of the *E6* and *E7* genes. Recently, microRNA heat map categorized by HPV infection and cervical cancer subtypes from TCGA projects also showed that the levels of miR-944, miR-767-5p, and miR-105-5p between HPV-positive and negative cancer were distinctively expressed [28]. Taken together, our results supported that miR-944 expression levels are closely related to HPV infection.

HPV E6 protein was found to bind p53 and inhibit cell apoptosis. HPV E7 proteins binding to retinoblastoma protein (pRb) interferes with cell cycle regulation [36, 37]. We found that miR-944 expression was associated with HPV E6/E7 mRNA. However, the relationship between miR-944 and p53 or pRb, which showed a relationship with HPV E6/E7, in cervical cancer progression was not evaluated. Further studies are needed to evaluate the functional role of miR-944 with p53 or pRb to understand the progression of cervical cancer.

The limitation of this study was the small sample size with single institution. Nevertheless, this study was valuable that miR-944 was shown potential marker complementing conventional clinical prognostic parameters and the relation to HPV E6/E7 positive cervical cancer tissues. It may be worthwhile to conduct replication to clarify our findings with sufficient clinical sample set.

## Conclusions

In conclusion, our results show that miR-944 was highly expressed in cervical cancer tissues and this was associated with FIGO stage, lymph node metastasis, and tumor size. Moreover, elevated expression of miR-944 was correlated with poor survival. Therefore, our data suggest that miR-944 could be used as a prognostic marker in cervical cancer.

## Additional files

Additional file 1: Relative expression of miR-944 in HPV-infected and HPV non-infected cervical cancer cell lines. The relative expression level of miR-944 was evaluated in five types of cervical cancer cell lines: C33A, SiHa, Caski, HeLa, and ME180. miR-944 was significantly up-regulated in SiHa (HPV 16), Caski (HPV 16), HeLa (HPV 18), and ME-180 (HPV 18, 68) cervical cancer cell lines compared to in the C33A (HPV-negative) cervical cancer cell line. (PPTX 74 kb)

Additional file 2: Clinical information from TCGA data. (PPTX 41 kb)

Additional file 3: miR-944 expression levels according to HPV E6/E7-positive vs. HPV E6/E7-negative cervical cancer in TCGA. The expression levels of miR-944 for 171 HPV E6/E7- positive cervical cancer patients including HPV α9 and HPV α7 species group and 9 HPV E6/E7- negative cervical cancer patients were analyzed. The expression level of miR-944 was significantly higher in the HPV E6/E7-positive cervical cancer patients than in the HPV E6/E7-negative cervical cancer patients (*P* < 0.0001). (PPTX 125 kb)

## Abbreviations

AUC: Area under the curve
cDNA: Complementary DNA
CI: Confidence interval
C_T_: Cycle threshold
DMEM: Dulbecco’s modified Eagle’s medium
FBS: Fetal bovine serum
FFPE: Formalin-fixed paraffin-embedded
FIGO: International Federation of Gynecology and Obstetrics
HPV: Human papillomavirus
HR-HPV: High-risk human papillomavirus
pRB: Retinoblastoma protein
ROC: Receiver operator characteristic
RT: Reverse transcriptase
RT-qPCR: Reverse transcription quantitative PCR
TCGA: The Cancer Genome Atlas
WHO: World Health Organization
