# Supplementary material for: microRNA-944 overexpression is a biomarker for poor prognosis of advanced cervical cancer
Source: BMC Cancer. 2019 May 6;19:419. doi: 10.1186/s12885-019-5620-6 (PMC6501303; doi:10.1186/s12885-019-5620-6)
Supplement: Supplementary file 1 — Relative expression of miR-944 in HPV-infected and HPV non-infected cervical cancer cell lines. The relative expression level of miR-944 was evaluated in five types of cervical cancer cell lines: C33A, SiHa, Caski, HeLa, and ME180. miR-944 was significantly up-regulated in SiHa (HPV 16), Caski (HPV 16), HeLa (HPV 18), and ME-180 (HPV 18, 68) cervical cancer cell lines compared to in the C33A (HPV-negative) cervical cancer cell line. (PPTX 74 kb) [file 12885_2019_5620_MOESM1_ESM.pptx]

## Slide 1
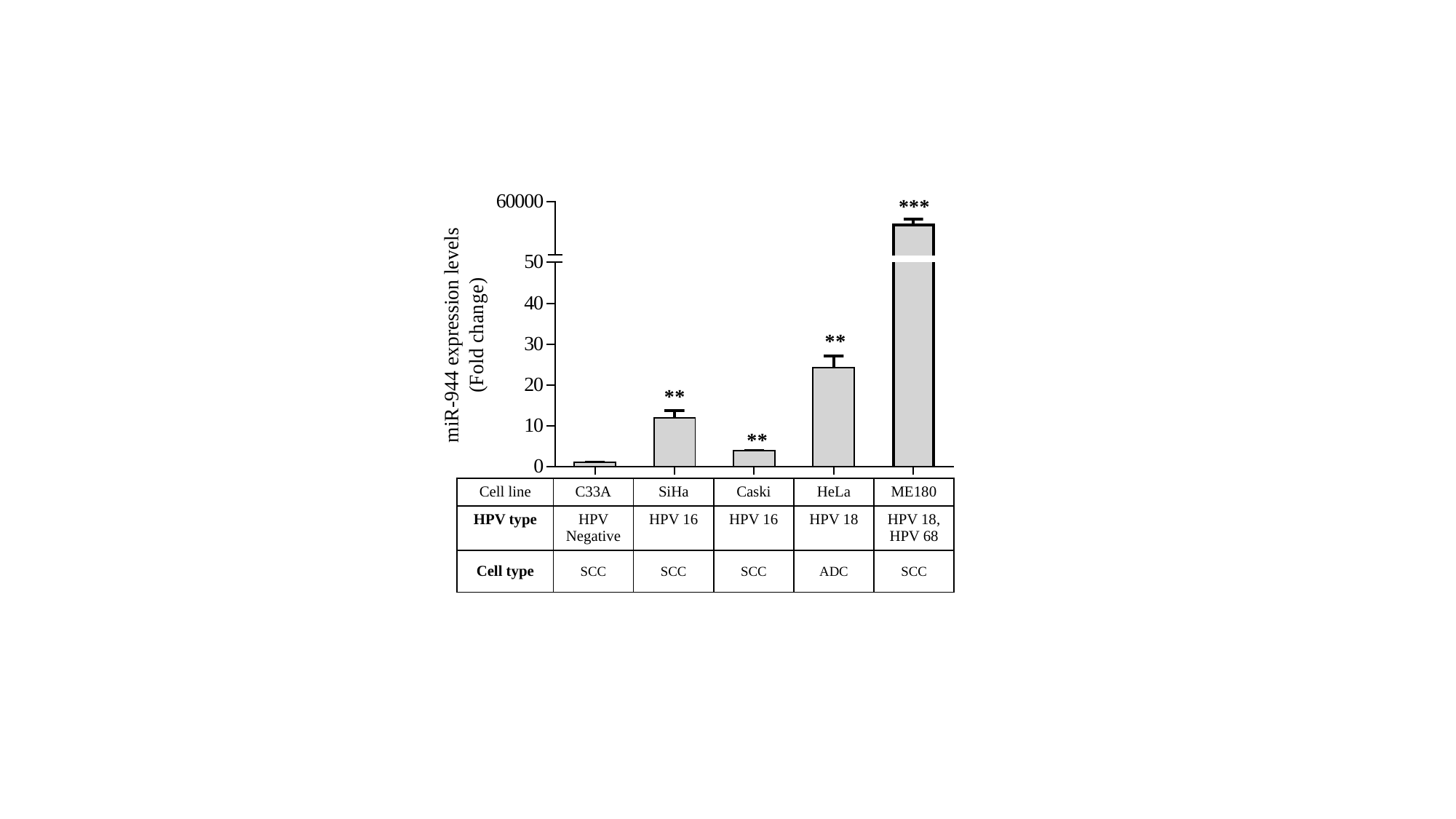

| Cell line | C33A | SiHa | Caski | HeLa | ME180 |
| --- | --- | --- | --- | --- | --- |
| HPV type | HPV Negative | HPV 16 | HPV 16 | HPV 18 | HPV 18, HPV 68 |
| Cell type | SCC | SCC | SCC | ADC | SCC |
