# Supplementary material for: microRNA-944 overexpression is a biomarker for poor prognosis of advanced cervical cancer
Source: BMC Cancer. 2019 May 6;19:419. doi: 10.1186/s12885-019-5620-6 (PMC6501303; doi:10.1186/s12885-019-5620-6)
Supplement: Supplementary file 2 — Clinical information from TCGA data. (PPTX 41 kb) [file 12885_2019_5620_MOESM2_ESM.pptx]

## Slide 1
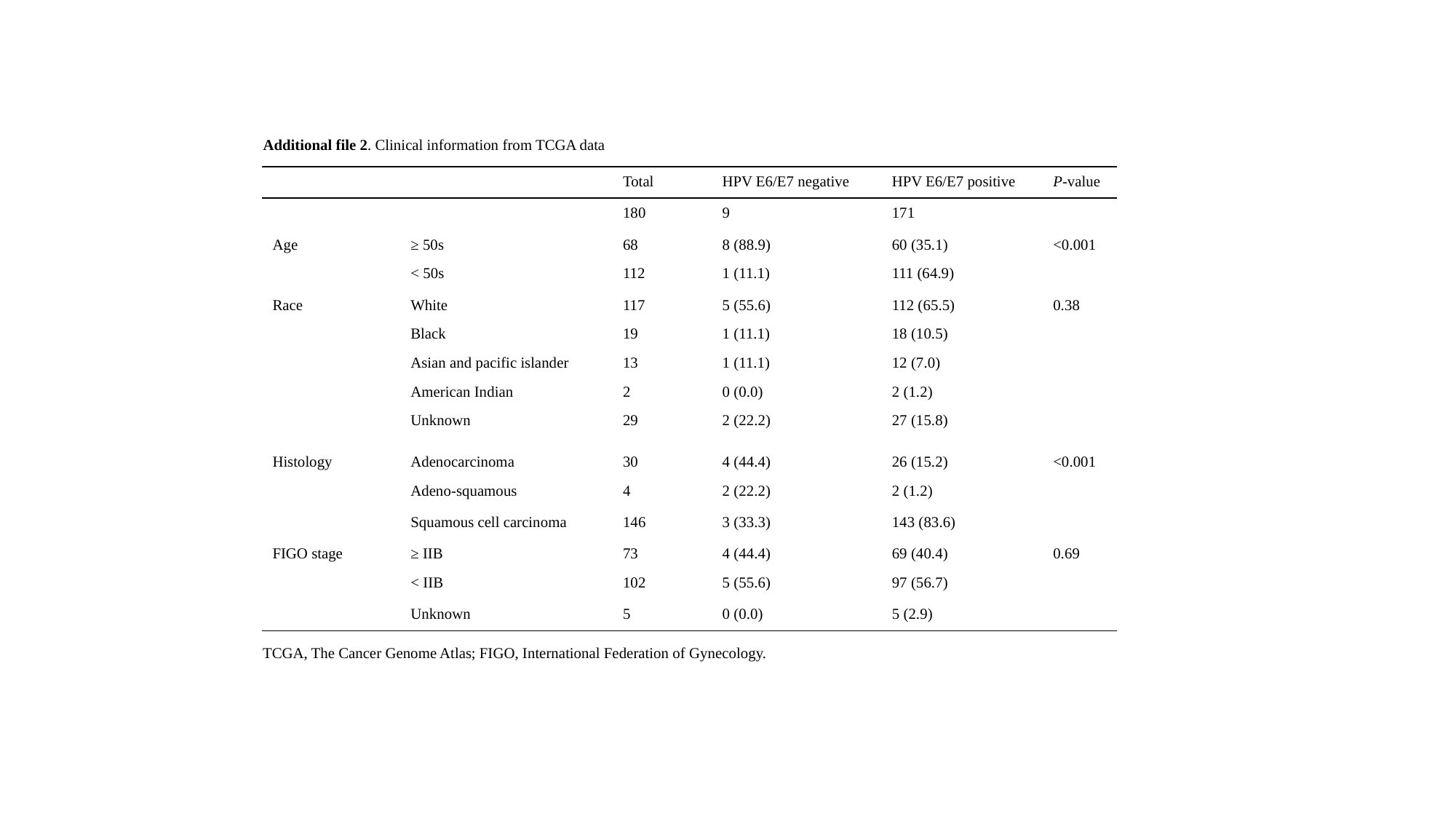

Additional file 2. Clinical information from TCGA data
| | | Total | HPV E6/E7 negative | HPV E6/E7 positive | P-value |
| --- | --- | --- | --- | --- | --- |
| | | 180 | 9 | 171 | |
| Age | ≥ 50s | 68 | 8 (88.9) | 60 (35.1) | <0.001 |
| | < 50s | 112 | 1 (11.1) | 111 (64.9) | |
| Race | White | 117 | 5 (55.6) | 112 (65.5) | 0.38 |
| | Black | 19 | 1 (11.1) | 18 (10.5) | |
| | Asian and pacific islander | 13 | 1 (11.1) | 12 (7.0) | |
| | American Indian | 2 | 0 (0.0) | 2 (1.2) | |
| | Unknown | 29 | 2 (22.2) | 27 (15.8) | |
| Histology | Adenocarcinoma | 30 | 4 (44.4) | 26 (15.2) | <0.001 |
| | Adeno-squamous | 4 | 2 (22.2) | 2 (1.2) | |
| | Squamous cell carcinoma | 146 | 3 (33.3) | 143 (83.6) | |
| FIGO stage | ≥ IIB | 73 | 4 (44.4) | 69 (40.4) | 0.69 |
| | < IIB | 102 | 5 (55.6) | 97 (56.7) | |
| | Unknown | 5 | 0 (0.0) | 5 (2.9) | |
TCGA, The Cancer Genome Atlas; FIGO, International Federation of Gynecology.
