# Supplementary figures and images for: microRNA-944 overexpression is a biomarker for poor prognosis of advanced cervical cancer
Source: BMC Cancer. 2019 May 6;19:419. doi: 10.1186/s12885-019-5620-6 (PMC6501303; doi:10.1186/s12885-019-5620-6)

## Slide 1
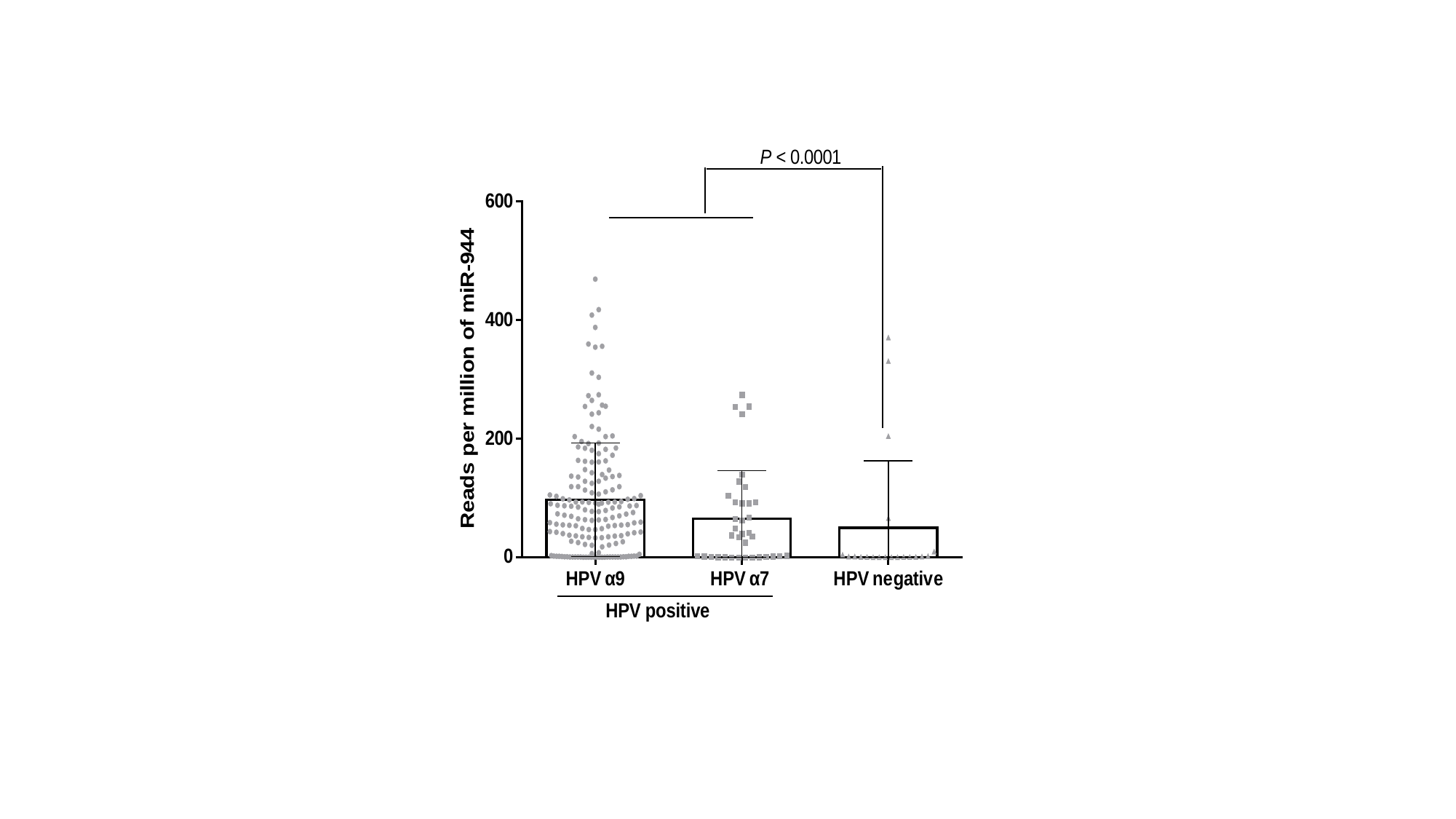

Supplement: Supplementary file 3 — miR-944 expression levels according to HPV E6/E7-positive vs. HPV E6/E7-negative cervical cancer in TCGA. The expression levels of miR-944 for 171 HPV E6/E7- positive cervical cancer patients including HPV α9 and HPV α7 species group and 9 HPV E6/E7- negative cervical cancer patients were analyzed. The expression level of miR-944 was significantly higher in the HPV E6/E7-positive cervical cancer patients than in the HPV E6/E7-negative cervical cancer patients (P < 0.0001). (PPTX 125 kb) [file 12885_2019_5620_MOESM3_ESM.pptx]
